# Sweeteners from Different Lingonberry Jams Influence on Bioaccessibility of Vitamin C, Anthocyanins and Antioxidant Capacity under In Vitro Gastrointestinal Digestion

**DOI:** 10.3390/antiox11030442

**Published:** 2022-02-23

**Authors:** Teodora Scrob, Anamaria Hosu, Claudia Cimpoiu

**Affiliations:** Department of Chemistry, Faculty of Chemistry and Chemical Engineering, Babeş-Bolyai University, 11 Arany Janos Street, 400028 Cluj-Napoca, Romania; teodora_scrob@yahoo.com (T.S.); anamaria.hosu@ubbcluj.ro (A.H.)

**Keywords:** lingonberries, vitamin C, anthocyanins, antioxidant capacity, bioaccessibility, in vitro digestion, sweeteners

## Abstract

Lingonberries are considered anot fully exploited major source of antioxidants. Their health benefits are closely linked to their bioavailability. Due to growing health concerns, consumers are looking for jams prepared with sweeteners other than white sugar, which could be a good alternative to meet their needs. The aim of this research was to evaluate the influence of sucrose, fructose, erythritol, brown sugar, coconut sugar, stevia and saccharine on the bioaccessibility of vitamin C, anthocyanins andthe antioxidant capacity of lingonberry jams under in vitro gastrointestinal digestion. The vitamin C, total anthocyanin content and antioxidant capacity measured by ABTS and FRAP assays were determined spectrophotometrically. Individual anthocyanins were determined by high performance liquid chromatography. All analyzed compounds were highly altered during gastrointestinal digestion and this effect was more visible in the case of the anthocyanins. Antioxidant capacity decreased after gastric digestion, but after the gastrointestinal step, radical scavenging capacity increased, while reducing power decreased. Vitamin C bioaccessibility was negatively affected by coconut sugar addition, while stevia addition showed a protective effect. Fructose and sucrose increased the total anthocyanin stability during the intestinal phase. Stevia, fructose and coconut sugar exhibited high protection of the antioxidant capacity of lingonberry jams during digestion.

## 1. Introduction

*Vaccinium vitis-idaea*, also commonly known as lingonberries, are small red berries of the Ericaceae family and the genus Vaccinium. They grow wild in the forests of northern countries, in Central Europe, Russia, and Canada [1,2]. These fruits are consumed raw or cooked in the form of lingonberry jam, compote, juice or syrup [3] and have been associated with antioxidant, anti-inflammatory and antimicrobial activities [2,3,4]. Lingonberries exhibit the highest antiproliferative properties among the berries [5] and have demonstrated their therapeutic potential both in vitro and in vivo [3]. Moreover, lingonberry intake has been associated with a beneficial effect on preventing and treating brain aging [2].

Lingonberries are classed as “superfruits”, being particularly rich in antioxidants such as anthocyanins [6], polyphenols, vitamins C, A, and E and functional compounds, such as fibers and minerals [1,2]. Anthocyanins, well-known natural alternatives to synthetic dyes, are the pigments of many flowers, fruits and vegetables [7] that have recently drawn much attention due to their numerous health-promoting properties [8]. In lingonberry fruits, three main anthocyanins, cyanidin-3-galactoside (main anthocyanin found in these berries), cyanidin-3-glucoside, and cyanidin-3-arabinoside, were identified [9]. The nutritional properties of lingonberry fruits are also related to the content of vitamin C, an essential nutrient that is of great importance tothe metabolism. It possesses great antioxidant power, eliminating free radicals, which minimizes damage to lipids, proteins and nucleic acids [10]. Vitamin C is a water-soluble vitamin and is not synthesized by the human organism [10]; therefore, it has to be obtained from the everyday diet.

Many fruits and vegetables, including lingonberries, are seasonal and perishable [7]. In this case, jam making is a good preservation technique for these fruits. Jams are most preferred by consumers mainly due to their availability, sensory quality and low cost. However, due to growing health concerns and the higher incidence of obesity, consumers are looking for high quality jams with low amount of sugar or products in which white sugar is substituted by other constituents [11]. Accordingly, the manufacture of diverse lingonberry jams sweetened with sweeteners other than white sugar may be a good alternative to fulfill these needs.

In order to evaluate the potential role of bioactive compounds in the human body, even if they are provided by fresh fruits or jams, changes occurring in the gastrointestinal (GI) tract should be taken into account [7]. During GI digestion, some bioactive compounds can be degraded and these structural changes could influence their bioactivity [12]. Anthocyanins, for consumers that eat berries on a routine basis, are major dietary components. However, pH and physiological temperatures may contribute significantly to the low bioavailability of anthocyanins, which does not justify all the biological activities previously associated with the huge consumption of this flavonoid class. Extensive knowledge of the amount of anthocyanins released in the GI tract and then absorbed is thus essential in understanding their health effects [13]. Vitamin C is another compound reported to be sensitive under digestion conditions. The alkaline pH and some factors inherent to in vitro GI digestion, such as temperature, oxygen and the enzyme activity, could enhance vitamin C oxidation or complex formation with other constituents [14]. Therefore, investigating anthocyanins and vitamin C stability in the GI tract could deliver important information on their bioavailability and their health benefits [8].

For this purpose and in order to simulate the GI digestion, in vitro methods are being used, since they are fast, simple and cheap [15]. Although in vivo studies provide more specific information about the bioavailability of bioactive compounds, in vitro digestion models are considered valuable and useful techniques for estimating the bioaccessibility of these compounds from foods [16].

The novel part of this study does not only consist of evaluating the changes in vitamin C, anthocyanins and the antioxidant capacity (AC) of lingonberry jams, but also of evaluating the changes in target compounds during in vitro GI digestion. Despite extensive research on the properties of lingonberry beverages, studies investigating the effect of GI digestion on dietary bioactive compounds such as vitamin C, anthocyanins or other antioxidant compounds are still scarce. Therefore, the aim of the present research was to investigate the influence of both natural and synthetic sweeteners (white sugar, fructose, erythritol, brown sugar, coconut sugar, stevia, saccharine) and in vitro GI digestion on vitamin C, individual and total anthocyanin content (TAC) and AC evaluated by ABTS and FRAP assaysof lingonberry jams. As far as we know, this is the first study which has focused on evaluating the changes of bioactive compounds in lingonberry jams under in vitro simulated digestion. 

## 2. Materials and Methods 

### 2.1. Chemicals

Ethanol 96%, 2,2′-Azino-bis(3-ethylbenzothiazoline-6-sulfonic acid) diammonium salt (ABTS), 2,4,6-tripyridyl-s-triazine (TPTZ), FeCl_3_·6H_2_O,Folin–Ciocalteu reagent, ascorbic acid, CH_3_COONa, 36.5% HCl, glacial acetic acid, H_2_SO_4_ (0.1 N), KMnO_4_, KCl, NaHCO_3_, KH_2_PO_4_, (NH_4_)_2_CO_3_, NaCl, MgCl_2_⋦(H_2_O)_6_ were purchased from Merck. α-Amylase, pepsin from porcine gastric mucosa, pancreatin from porcine pancreas, bile salts were purchased from Alfa Aesar (Karslruhe, Germany). All reagents used in the experimental part were of analytical purity. Sweeteners used in this study were purchased from a health food store and local market.

### 2.2. Jam Preparation

Fresh lingonberries (*Vaccinium vitis-idaea* L.) were harvested from Apuseni Mountains, Romania, in August, 2019. Lingonberry jams were prepared in the laboratory. The recipe followed a traditional and easy procedure [11]. All jams were prepared using 100 g of fruit and the following quantities of sweeteners: 50 g white sugar (Jam 1), 29.4 g fructose (Jam 2), 77.0 g erythritol (Jam 3), 50.0 g brown sugar (Jam 4), 50.0 g coconut sugar (Jam 5), 0.180 g stevia (Jam 6) and 0.180 g saccharine (Jam 7). A low temperature (50 °C) was chosen to heat the ingredients in order to prevent the degradation of sensitive compounds, such as anthocyanins [11]. Heatingprocess was stopped when total soluble content (TSS) reached value of 56–57° Brix [7]. In the case of jams 6 and 7, heating was stopped when TSS reached about 22° Brix, as previously reported by Sutwal et al. [17]. Each jam was packed into glass jars without being pasteurized and were kept in the freezer until analysis.

The jam extracts were prepared as follows: 1.0 g of each jam mixed with 10 mL 45% ethanol was stirred for 1 h at room temperature. The mixtureswere then sonicated at 30 °C for 30 min in a thermostatic bath Elmasonic E60H (Elma Schmidbauer GmbH, Singen am Hohentwiel, Germany). The extracts were centrifuged at 875 g for 20 min using a Centurion Scientific centrifuge C2006 (Centurion Scientific Limited, Bosham, UK). Each extract was filtered out and the supernatants were collected and used for further analyses.

### 2.3. In Vitro Simulated GI Digestion

GI digestion was performed according to the protocol developed by Minekus [15]. Salivary, gastric and intestinal digestions were sequentially simulated in this study. Briefly, the simulated digestion was conducted with 5 g of each jam sample. The salivary digestion was simulated using α-amylase solution and simulated salivary fluid. For gastric digestion step (pH 3.0), pepsin and simulated gastric fluid were added to the salivary step, the mixture was temperature-controlledat 37 °C and stirred for 2 h. For simulation of intestinal digestion (pH 7.0), pancreatin, bile salts and simulated intestinal fluid were added and the digestion mixture was stirred again for 2 h at 37 °C. Both gastric and intestinal samples were centrifuged 10 min at 875 g and the supernatants were collected for further analyses.

The bioaccessibility (%) of vitamin C, TAC and AC from each jam was calculated by following formula [18].

Bioaccessibility (%) = (C_digestion_/C_undigested_) × 100 (1)

where, C_digestion_ is the concentration of the compound after the G or GI digestion and C_undigested_ is the concentration of the compound in the undigested sample.

### 2.4. High Performance Liquid Chromatography (HPLC) Analysis of Anthocyanins

For the analysis of anthocyanins from lingonberry fruits and jams, the method of Zheng et al. [19] was applied with some modifications. Samples were 2-fold diluted with 5% formic acid, passed through 0.2µm membrane filters and then 20 µL was injected into an Agilent 1200 HPLC system (Agilent Technologies Inc.; Santa Clara, CA, USA) equipped with a diode array detector. An Eclipse XTB-C18 (Agilent) column (150 × 4.6 mm inner diameter, particle size 5µm) was used as stationary phase. The mobile phase consisted of 5% aqueous formic acid (A) and HPLC grade acetonitrile (B). The flow rate was 1 mL/min, with a gradient profile as follows: 0–1 min, 4% B; 1–10 min, 4–6% B; 10–15 min, 6% B; 15–35 min, 6–18% B. The detection was performed at 532 nm and the anthocyanins were identified by comparing with standards based on the retention times.

### 2.5. Spectrophotometric Measurements

A T80+ UV–Vis spectrophotometer was used for the spectrophotometric measurements. Each sample was analyzed in triplicate at room temperature.

#### 2.5.1. Vitamin C Determination

The determination of vitamin C was performed according to Zanini et al. [10]. Aliquots of 2.0 mL of each extract optimally diluted were mixed with 2.0 mL of 0.1 mol/L KMnO_4_ prepared in H_2_SO_4_. The absorbance was immediately read at 525 nm. Ultrapure water was used instead of sample for blank preparation. The decrease of the absorbance (ΔA) was calculated using the following equation:

ΔA = A_blank_ − A_sample_ (2)

The content of vitamin C was obtained from the calibration curve achieved in the same conditions and was expressed as mg/g jam. 

#### 2.5.2. Total Anthocyanin Content (TAC) Determination

TAC in the extracts and digested samples was determined by pH-differential method, according to Moldovan et al. [20]. Aliquots of 1 mL of each sample were 4-fold diluted with KCl/HCl buffer, 0.025 M, pH = 1 and CH_3_COONa/CH_3_COOH buffer, 0.4 M, pH = 4.5. The absorbance of each solution was measured at 532 nm and at 700 nm after 15 min. TAC was calculated using Equation (2):

TAC = (A × MW × DF × 1000) (ε × l)(3)

where MW = molecular weight (449.2 g/mol); DF = dilution factor; l = path length (1 cm); ε = molar extinction coefficient (26,900 L/mol·cm); 1000 = conversion factor from gram to milligram. The absorbance (A) was calculated by Equation (3):

A = (A_pH 1.0_ − A_pH 4.5_)_532nm_ − (A_pH 1.0_ − A_pH 4.5_)_700nm_ (4)

TAC was expressed as cyanidin-3-glycoside equivalents (mg Cy-3-gly/g jam). 

#### 2.5.3. Antioxidant Capacity (AC) Determination

The radical scavenging capacity (AC_ABTS_) of samples was determined using the ABTS assay [21]. ABTS^•+^ solution was previously prepared by mixing 1:1 ABTS solution (7 mM) and K_2_S_2_O_8_ solution (2.45 mM). Before being used, the solution was kept in the dark at room temperature for 24 h. Prior to analyses, the ABTS^•^^+^ solution was diluted till it had an absorbance of 0.8–0.9 at 734 nm. Aliquots of 0.5 mL of diluted sample was mixed with 3 mL ABTS^•^^+^ solution. The absorbance of each sample was read at 734 nm after 15 min. The AC was calculated on the basis of the calibration curve and expressed as Trolox equivalents (μmols/g jam).

Ferric reducing antioxidant power (AC_FRAP_) of jams before and after the in vitro digestion was carried out according to the procedure described by Hosu et al. [22]. Briefly, the FRAP reagent was prepared using 2.5 mL of 10 mM 2,4,6-tripyridyl-s-triazine (TPTZ) solution in HCl, 25 mL 0.3 M acetate buffer (pH = 3.6) and 2.5 mL of 20 mM ferric chloride. FRAP reagent was freshly prepared and warmed in a water bath at 37 °C before use. Optimally diluted samples (150 μL) were added to 2850 μL of the FRAP working solution and incubated in the dark for 30 min. The reduction of the Fe^3+^-TPTZ complex was monitored by measuring the absorbance at 593 nm. Ascorbic acid was used for the calibration curve and the results were expressed as ascorbic acid equivalents (mg vitamin C/g of jam).

### 2.6. Statistics

The results are presented as mean ± standard deviations of three replicates. Analysis of variance (ANOVA), Student’s *t* tests and correlation tests were performed by means of StatistiXL for Excel. The difference was considered statistically significant at a level of *p* < 0.05 for probability of 95%.

## 3. Results and Discussion

### 3.1. Bioaccessibility of Vitamin C

The highest vitamin C concentration of non-digested jams was found in jam prepared with stevia (15.5 mg/g jam), followed by jam formulated with saccharine and fructose (13.6 mg/g jam and 13.4 mg/g jam, respectively) (Table 1). Erythritol-based jam presented the lowest concentration of vitamin C before digestion (9.92 mg/g jam).

**Figure 1:**
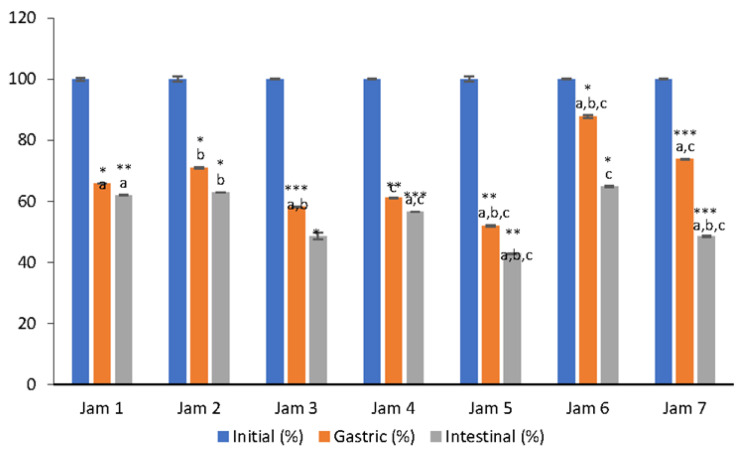
Changes in bioaccessibility (%) of vitamin C during in vitro simulated digestion of lingonberry jams. Data represent average values ± standard deviation of three independent measurements and initial bioaccessibility determined in non-digested jams is set as 100%. Letters a,b,c indicate statistically significant differences between the values for each phase of digestion (*p* < 0.05) according to Student’s *t* tests. Asterisk symbols signify the following levels of statistical significance of differences according to one way ANOVA and Student’s *t* tests: *** *p* < 0.0001, ** *p* < 0.001, * *p* < 0.05.

The GI bioaccessibility of vitamin C from lingonberry jams ranged from 42.8% to 62.9% (Figure 1). The vitamin C concentration was reduced to less than 50% in the gastric phase, indicating a higher stability of this compound under acidic conditions. The acidic conditions of the gastric environment could protect the vitamin C against its chemical or enzymatic oxidation [14]. Moreover, other authors demonstrated that gastric digestion had little effect on vitamin C stability, recovering 71% of this bioactive compound in pomegranate juice [23]. However, differences in gastric bioaccessibility was observed between lingonberry jams, indicating that the type of sweetener used could influence the stability of vitamin C. Overall, the bioaccessibility of vitamin C was higher in jams formulated with stevia (87.7%) and saccharine (73.7%) compared to erythritol (58.2%) and coconut sugar (51.9%) based jams. A higher bioaccessibility of vitamin C in the presence of stevia could be attributed to the protective effect of stevioside on the degradation of ascorbic acid, as reported by Kroyer [24]. The vitamin C concentration decreased in the intestinal phase compared to the gastric phase. These results show that vitamin C is more unstable under intestinal conditions. The alkaline pH and other digestion factors, such as temperature or enzyme activity may enhance the vitamin C oxidation or complex formation with other constituents [14].

Overall, the type of sweetener used in jam formulation had a significant influence on the bioaccessibility of vitamin C (*p* = 0.0001), the highest GI bioaccessibility of vitamin C being observed in stevia jam (64.9%) (Figure 1).

The high bioaccessibility of vitamin C in the presence of stevia could probably be attributed to the synergistic interactions between the vitamin C and other constituents present in this natural sweetener, such as stevioside [24]. In contrast, the lowest level of vitamin C (42.8%) was found after GI digestion of coconut sugar-based jam, but there is no study published to date showing possible interferences between this sweetener and ascorbic acid.

These results suggest that almost half of the total vitamin C content became bioaccessible and could be absorbed in the GI tract. Lingonberries are rich in antioxidants and polyphenols [1,2] and it could be predicted that these compounds may prevent vitamin C oxidation and degradation during digestion [25]. However, there are no literature reports concerning the sweetener effect on the bioaccessibility of vitamin C from lingonberry jams or derivates. Cilla et al. [26] reported a high vitamin C bioaccessibility (between 44% and 83.7%) in grape, peach and apricot blended fruit juices. A higher bioaccessibility of vitamin C in previous research may be due to the food matrix composition [14]. Taking into account the behavior of vitamin C in the presence of different sweeteners, jam formulation may affect the bioaccessibility of the bioactive compounds [25].

### 3.2. Bioaccessibility of Anthocyanins 

Anthocyanins are generally instable compounds andtheir stability can be influenced by various factors, such as pH, oxygen, presence of enzymes, etc. [8]. Consequently, the determination of their bioaccessibility after passing through the gastrointestinal tract is very important. To this end, the behavior during in vitro GI digestion of both total and individual anthocyanins was investigated.

**Figure 2:**
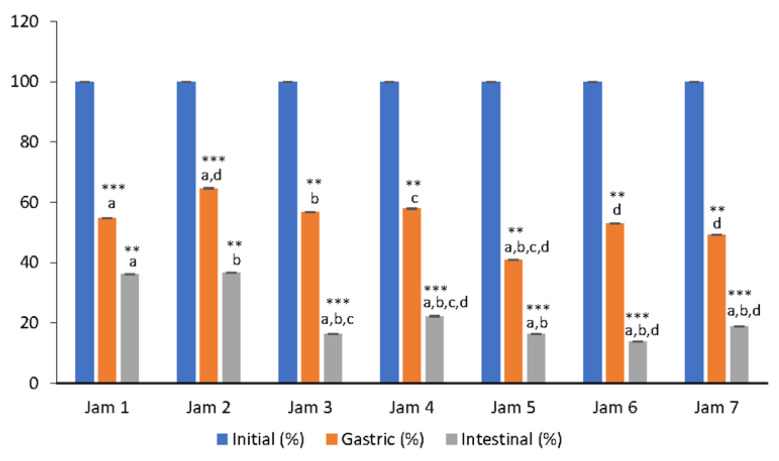
Changes in bioaccessibility(%) of total anthocyanins during in vitro simulated digestion of lingonberry jams. Data represent average values ± standard deviation of three independent measurements and initial bioaccessibility determined in non-digested jams is set as 100%. Letters a,b,c,d indicate statistically significant differences between the values for each phase of digestion (*p* < 0.05) according to Student’s *t* tests. Asterisk symbols signify the following levels of statistical significance of differences according to one way ANOVA and Student’s *t* tests: *** *p* < 0.0001, ** *p* < 0.001.

The highest TAC of non-digested jams was found in jams prepared with saccharine and stevia (0.263 mg/g jam and 0.261 mg/g jam, respectively), while erythritol based jam presented the lowest TAC (0.164 mg/g jam) before digestion (Table 1). The variations in the amount of bioaccessible anthocyanins after gastric and GI digestion are shown in Figure 2.

It was observed that the total anthocyanins were stable under gastric conditions sincemost of the jam samples hada TAC bioaccessibility higher than 50%. These results are in agreement with those obtained by others [27,28], which claimed that 75–88% of the total anthocyanins could be recovered from the gastric conditions. The low pH in the stomach may contribute to the high stability of anthocyanins [8]. Moreover, their absorption through the stomach can be estimated by different models of gastric cells [28]. Therefore, total anthocyanins may exert their health benefits in the gastric phase of digestion, without being absorbed through the intestinal wall. It should also be noted that the anthocyanins’ bioaccessibility in the stomach was affected by the sweeteners used in the lingonberry jams. For instance, fructose exhibited the highest protection upon TAC degradation (bioaccessibility of 64.6%), while the gastric bioaccessibility of anthocyanins was decreased to 40.9% in the presence of coconut sugar. Lower levels of anthocyanins in the presence of coconut sugar might be due to the antagonistic interactions between this sweetener and anthocyanins, but there are no reported results in this sense. However, as far as we know, no literature studies regardingthe in vitro bioaccesibility of anthocyanins in the presence of natural or synthetic sweeteners were found.

The transition to the intestinal phase caused a significant decrease (*p* < 0.05) in the bioaccessibility of total anthocyanins. The lowest bioaccessibility of TAC at the end of GI digestion was observed in the case of stevia-based jam (13.7%), while fructose showed a protective effect upon the TAC bioaccessibility (36.5%) (Table 2).

The low recovery of anthocyanins could be partially due to the low stability of these compounds under alkaline conditions (pH = 7.5) [8]. This might be attributed to the structural changes of the flavylium cation to a colorless, less stable chalcone [29]. Our results are in good agreement with study of David et al. [8] that reported a decrease of Cornelian cherries’ anthocyanins by more than 70% compared to the undigested sample. A remarkable impact of intestinal digestion on TAC was also reported by Betanzo et al. [30], where a decrease of blueberry anthocyanins, higher than 80%, was found. The reason for the high loss of anthocyanins remains not quite clear. However, it is evident that the food matrix may play an important role in anthocyanin behavior during digestion, since these compounds exhibit different bioaccessibility levels in the presence of different sweeteners.

**Figure 3:**
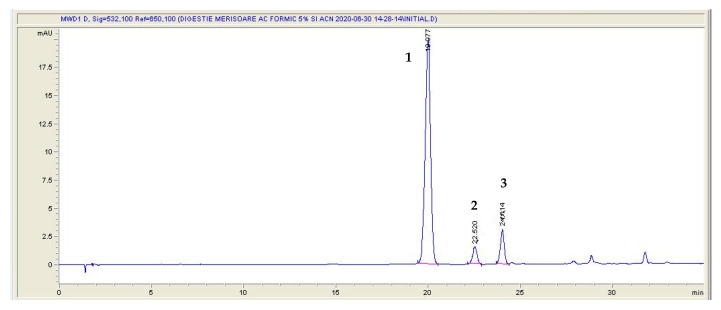
HPLC profile (532 nm) of anthocyanins: (**1**) cyanidin 3-galactoside; (**2**) cyanidin 3-glucoside; (**3**) cyanidin 3-arabinoside.

The individual anthocyanins were analyzed in lingonberry jam extracts by HPLC. Three main anthocyanins were identified by comparing the retention times with those obtained for standards and these were assigned to cyanidin 3-galactoside (Peak 1), cyanidin 3-glucoside (Peak 2) and cyanidin 3-arabinoside (Peak 3) (Figure 3). These results are in agreement with those reported by Zheng et al. [19] that also identified these anthocyanins in the Canadian lingonberry.

Among the anthocyanin constituents of lingonberry jams, cyanidin 3-galactoside stands out as being the most abundant anthocyanin (84.61% from total anthocyanin content). Cyanidin 3-galactoside is one of the most widespread anthocyanins that positively impact on the health of animals and humans. Its antioxidant properties and other health effects, including anti-inflammatory, antidiabetic or nervous protective capacities make this compound a potentially valuable food additive or health supplement [31]. Therefore, investigating what happens to this compound after ingestion in the presence of different sweeteners could be of a real importance for the food industry. In addition to cyanidin 3-galactoside, cyanidin 3-arabinoside and cyanidin 3-glucoside were identified in lower amounts (9.76% and 5.63%, respectively). 

**Figure 4:**
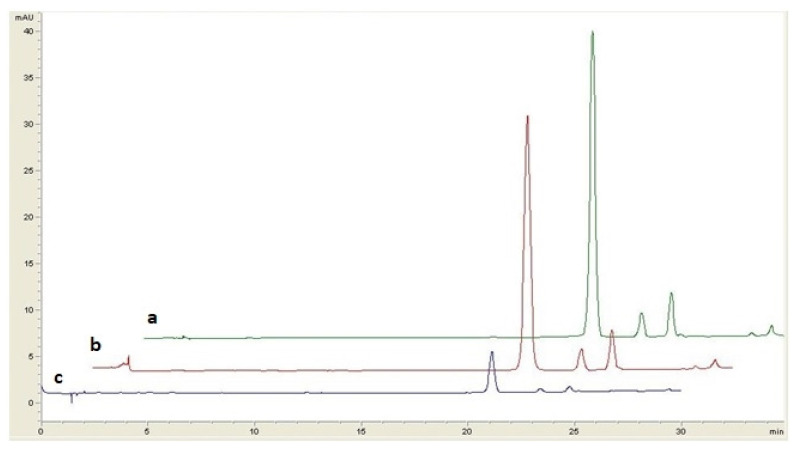
HPLC profiles (532 nm) of anthocyanins from stevia lingonberry jam before (**a**) and after in vitro gastric (**b**) and GI (**c**) digestion.

During the in vitro GI digestion, the individual anthocyanins followed a similar behavior with that of TAC, with very good correlation coefficients of 0.9691, 0.9548 and 0.9730, respectively. After gastric digestion, the bioaccessibility of the lingonberry jams’anthocyanins was higher than 50%, except for jams prepared with coconut sugar, stevia and saccharine (Table 2). Following the GI phase, the three anthocyanins presented a higher decrease than in the gastric phase, indicating the sensitivity of these compounds under this digestion phase (Table 2). GI digestion determined low bioaccessibility of individual anthocyanins (<30%), except for the fructose- and sucrose-based jams, where anthocyanins’ bioaccessibility ranged between 41.8% and 56.4%. Changes in individual anthocyanin concentrations during GI digestion were exemplified by HPLC chromatograms in the case of stevia-based jam (Figure 4).

The sweetener used in each jam formulation had a significant effect (*p* < 0.05) on each anthocyanin’s GI bioaccessibility, influencing the possible absorption of these compounds. Following the gastric digestion step, the bioaccessibility of cyanidin-3-galactoside in the sucrose-based jam was 69.2%, while stevia determined a lower bioaccessibility of this compound (46.7%). Cyanidin-3-glucoside presented a very low bioaccessibility (<10%) under small intestine conditions in the presence of saccharine, coconut sugar and stevia jam, while in the presence of sucrose and fructose, its bioaccessibility ranged between 43.8% and 46.1%, respectively. Fructose obviously protected the cyanidin-3-arabinoside, its GI bioaccessibility being 56.3%in the presence of this sweetener, while saccharine and stevia determined the lowest recoveryof this anthocyanin during the intestinal step, 8.7% and 8.3%, respectively. The results show that cyanidin-3-galactoside degradation was greater during in vitro digestion compared to that of cyanidin-3-glucoside. This fact may be due to the hexoside moiety of anthocyanin. It was reported that the glucoside moiety is more stable than the galactoside moiety during digestion [32].

### 3.3. Changes in AC of Lingonberry Jams during In Vitro Digestion

Health benefits of lingonberries are partly attributed to their AC [3]. Due to the fact that each method of assessing AC is based on a different reaction mechanism, at least two assays must be used for its determination [33]. Therefore, the ABTS assay and FRAP assay were used to assess the AC of jams both on non-digested and in vitro digested samples.The AC_ABTS_ of non-digested lingonberry jam extracts ranged from 66.6 µmol Trolox/g of jam to 109 µmol Trolox/g of jam, while AC_FRAP_ varied from 4.64 mg vit C/g of jam to 9.37 mg vit C/g of jam(Table 1). Regardless of the method used, stevia-based jam has the highest AC, followed by saccharine- and coconut sugar-based jams, and erythritol-based jam exhibited the lowest AC value. 

**Figure 5:**
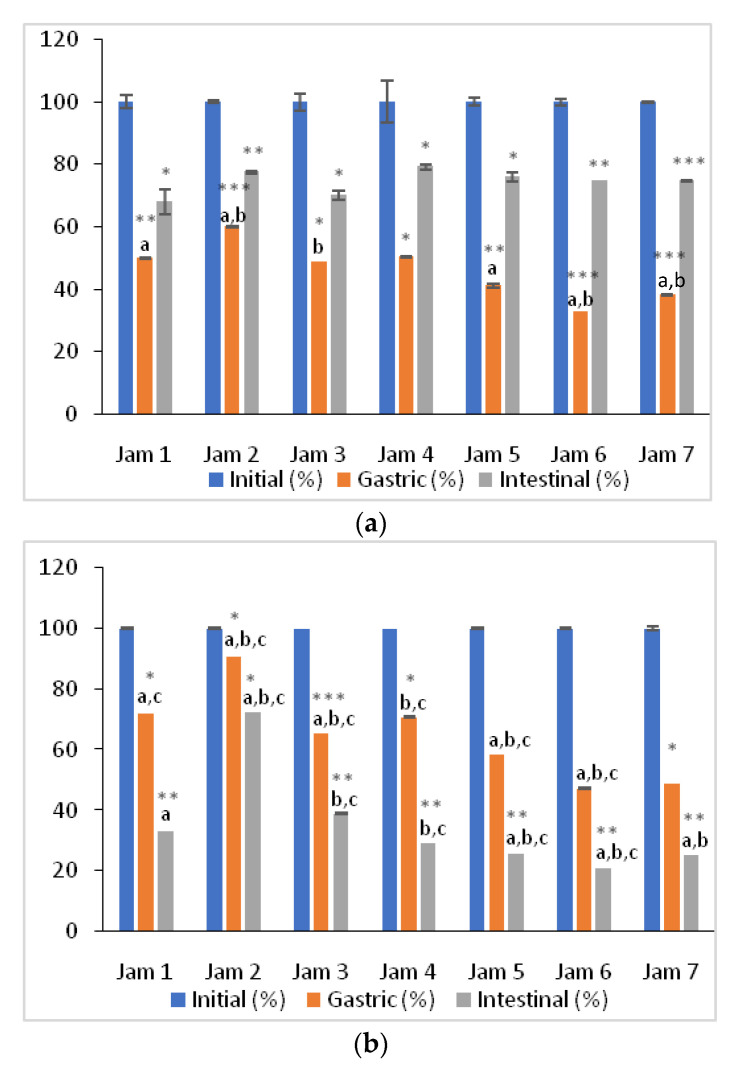
Changes in bioaccessibility(%) of antioxidants by ABTS (**a**) and FRAP (**b**) assays during in vitro simulated digestion of lingonberry jams. Data represent average values ± standard deviation of three independent measurements and initial bioaccessibility determined in non-digested jams is set as 100%. Letters a,b,c indicate statistically significant differences between the values for each phase of digestion (*p* < 0.05) according to Student’s *t* tests. Asterisk symbols signify the following levels of statistical significance of differences according to one way ANOVA and Student’s *t* tests: *** *p* < 0.0001, ** *p*< 0.001, * *p* < 0.05.

Following the gastric phase ofthe in vitro digestion model, the ACs of all lingonberry jams significantly decreased (*p* < 0.05) in the case of both methods used (Figure 5), the highest decrease being observed in the AC_ABTS_. During this step of digestion, the lowest bioaccessibility of antioxidants was obtained in the case of stevia jam (32.8% and47.3%, respectively), while the fructose jam show the highest bioaccessibility (59.9% and 90.8%, respectively). The AC_ABTS_ of all the digested samples significantly increased (*p* < 0.05) during the transition from the gastric to the intestinal environment (Figure 5a). Contrarily, the AC_FRAP_ values significantly decreased (*p* < 0.05) in the GI phase of digestion in all tested samples (Figure 5b).The results suggest that the antioxidant compounds present in lingonberry jams are capable of both scavenging free radicals and reducing oxidants at the gastric level. However, at the end of GI digestion, the antioxidant capacity of lingonberry jams might be due rather to the antioxidants capable of scavenging radicals.

In the case of ABTS assay, the increasein bioaccessibility values from the gastric to the intestinal phase varied between 2.27-fold in the case of stevia jam and by 1.29-fold in the case of fructose jam. It could be hypothesized that the structural changes that may accompany the outcome of anthocyanin digestion might directly contribute to the AC_ABTS_ of the digestion samples [29]. Thus, it is known that under the acidic conditions in the stomach, anthocyanins have been chemically hydrolyzed and degraded to yield phenolic acids [26,32]. Despite the decrease of TAC in GI digestion, there was not observed a similar behavior of the AC_ABTS_. The high bioaccessibility values in the intestinal phase might be due to the presence of substances other than anthocyanins or due to the anthocyanin degradation products, compounds that possess AC. Similar to our results, Tagliazucchi et al. [34] demonstrated a significantly higher radical-scavenging activity (ABTS) of grapes during the transition from the acidic gastric to the mild alkaline intestinal environment.A similar trend was also observed in another study [35].The radical scavenging activities in the intestinal phase are even higher than those exhibited before digestion in a study reported by Fawole&Opara [35], presumably due to the dependency of phenolicactivity on the pH of the digestion medium. It was reported that alkaline pH significantly increases phenolic scavenging ability [34].

In comparison to AC_ABTS_, AC_FRAP_ decreased significantly following GI digestion and the highest decline in the antioxidants’ bioaccessibility was observed in the case of stevia-based jam (Figure 5b). Decreases in AC_FRAP_ values following GI digestion was also reported by Fawole & Opara [34] in a study on pomegranate co-products. Chen et al. [36] also found a significant decrease in AC_FRAP_ valuesafter the gastric and duodenal phases of digestion in a study on plums. The observed decrease of the reducing power of theinvestigated jams could primarily be due to the pHof the medium [35]. The pH of a substance is known to affect racemization of molecules, and this fact may conduct to two chiralenantiomers with different reactivity [37]. Therefore, some antioxidants could be rendered more reactive atacidic pH in the gastric phase and less reactive at alkalinepH during the intestinal phase of in vitro digestion [35,37], a trend observed in our research regarding AC_FRAP._ Furthermore, it could also be suggested that metabolites formed as a result of structural changes in the alkaline condition could have reacted differently in the FRAP assay [35,37]. A good correlation was observed between TAC and AC_FRAP_ values recorded during GI digestion, Pearson’s correlation coefficient being 0.9088, a correlation that is not valid for TAC and AC_ABTS_ values (r = 0.2530). These results suggest anthocyanins by their metal-chelating activity exhibit the protecting ability against formation of hydroxyl radicals. The effect of the sweeteners on the AC_ABTS_ and AC_FRAP_ of the lingonberry jams was also evaluated, with a significant influence (*p* < 0.05) on the bioaccessibility of antioxidants after in vitro GI digestion (Figure 5). It should be noted that in the case of AC_ABTS_ after GI digestion no statistically significant differences (*p* > 0.05) were observed between jams. The presence of fructose exhibited the highest bioaccessibility of antioxidants at gastric level by both used assays, indicating a protective effect of this sweetener at this stage of digestion. In contrast, stevia-based jam presented an almost 2-fold lower bioaccessibility of ABTS scavenging antioxidants following the gastric step, but this sweetener brought on a very high bioaccessibility of antioxidants at the intestinal step. In a study of Bender et al. [38], the AC of red raspberry juice was significantly enhanced (*p* < 0.0001) with the addition of stevia. On the other hand, sucrose-based jam presented the lowest ABTS scavenging antioxidants’ bioaccessibility at the end of simulated digestion. This finding could be related to the fact that refined sweeteners are hypothesized to contain lower levels of antioxidants than unrefined products [39]. In our study, following in vitro digestion, unrefined sweeteners such as brown sugar and coconut sugar determined a higher AC_ABTS_ than white refined sugar. Therefore, alternatives to unrefined sugar should be used in jam formulation in order to protect the radical-scavenging antioxidant degradation during the complex digestion process. In contrast, antioxidants determined by FRAP assay did not show a higher bioaccessibility in the presence of unrefined sugars.Although stevia-based jam presented the highest GI bioaccessibility of antioxidants using the ABTS method, the FRAP assay determined the lowest bioaccessibility of antioxidants after undergoing GI digestion. Saccharine-based jam also showed a very good radical scavenging capacity and a poor reducing capacity (Figure 5). Further to this, our results suggest that AC generally depends on the antioxidants’ structure at different GI digestion levels.

## 4. Conclusions

The influence of sweeteners on the in vitro bioaccessibility of vitamin C, anthocyanins and AC from lingonberry jams were investigated in this research. The vitamin C concentration was reduced by less than 50% in the gastric phase, but its concentration decreased in the intestinal phase. GI digestion resulted in a high loss of TAC and all three anthocyanins found in lingonberry jams were quite stable under gastric conditions, but significantly decreased after the intestinal step. Remarkably, it was found that AC_ABTS_ was higher after the GI phase than after the gastric phase, indicating that antioxidant compounds are progressively released from the food matrix and can exert their radical scavenging activity and biological effects. In contrast, AC_FRAP_ decreased in the case of all samples after the GI digestion step, indicating that antioxidants from lingonberry jams have a higher radical scavenging ability than a reducing power. The results also demonstrated that the food matrix may play an important role in the stability of bioactive compounds during digestion. For instance, the highest GI bioaccessibility of vitamin C was reached in jam containing stevia. Fructose showed a positive effect upon anthocyanins during digestion. Stevia, fructose and coconut sugar exhibited high protection of the AC of lingonberry jams during digestion. Therefore, available alternatives to refined white sugar may be used in jam formulation, offering potential benefits. This study gives a detailed understanding of the changes in the bioactive compound content of lingonberry jams during digestion in the presence of different sweeteners and could make a valuable contribution in developing novel sources of healthy foods. 

## Figures and Tables

**Table 1:** Vitamin C content, TAC and AC of non-digested lingonberry jams.

Lingonberry Jam	Vitamin C (mg/g jam)	TAC (mg Cy-3-gly/g Jam)	AC (μmols Trolox/g Jam)	AC_FRAP_ (mg Vitamin C/g Jam)
Jam 1 (white sugar)	12.85 ± 0.49 ^a^	0.212 ± 0.001 ^a^	56.2 ± 2.0 ^a^	5.39 ± 0.10 ^a^
Jam 2 (fructose)	13.37 ± 0.85 ^b^	0.254 ± 0.002 ^a,b^	51.4 ± 0.4 ^b^	5.64 ± 0.08 ^b^
Jam 3 (erythritol)	9.92 ± 0.15 ^a,b^	0.164 ± 0.003 ^a,b,c^	45.3 ± 2.7 ^a^	4.64 ± 0.02 ^a,b,c^
Jam 4 (brown sugar)	12.50 ± 0.15 ^b,c^	0.214 ± 0.009 ^b,c,d^	57.3 ± 6.6 ^c^	5.36 ± 0.06 ^c^
Jam 5 (coconut sugar)	10.31 ± 0.89 ^d^	0.204 ± 0.007 ^b,c^	72.4 ± 1.3 ^a,b^	7.82 ± 0.10 ^a,b,c^
Jam 6 (stevia)	15.51 ± 0.15 ^a,b,c,d^	0.261 ± 0.027 ^c^	93.8 ± 1.1 ^a,b,c^	9.37 ± 0.23 ^a,b,c^
Jam 7 (saccharine)	13.57 ± 0.03 ^b,c,d^	0.263 ± 0.005 ^a,c,d^	72.1 ± 0.2 ^a,b^	8.85 ± 0.68 ^a,b,c^

Values represent mean ± SD (*n* = 3). Superscripts (a,b,c,d) indicate statistically significant differences (*p* < 0.05) according to Student’s *t* tests.

**Table 2:** Bioaccessibilities of TAC and individual anthocyanins: cyanidin-3-galactoside (cy-3-gal), cyanidin-3-glucoside (cy-3-glu) and cyanidin-3-arabinoside (cy-3-ara) during in vitro simulated digestion of lingonberry jams, expressed as %.

		Gastric Digestion				GI Digestion			
	Anthocyanins	TAC	Cy-3-gal	Cy-3-glu	Cy-3-ara	TAC	Cy-3-gal	Cy-3-glu	Cy-3-ara
Jam									
**Jam 1**		54.8 ^a^	69.2 ^a^	72.9 ^a^	65.9 ^a^	36.1 ^a^	45.1 ^a^	46.1 ^a^	41.8 ^a^
**Jam 2**		64.6 ^a,d^	61.3 ^a,d^	65.9 ^a,d^	66.6 ^a,d^	36.5 ^b^	47.5 ^b^	43.8 ^b^	56.4 ^b^
**Jam 3**		56.7 ^b^	68.9 ^b^	74.5 ^b^	66.9 ^b^	16.4 ^a,b,c^	27.7 ^a,b,c^	30.1 ^a,b,c^	25.9
**Jam 4**		57.9 ^c^	60.2 ^c^	63.7 ^c^	57.2 ^c^	22.2 ^a,b,c,d^	26.2 ^a,b,c,d^	29.1 ^a,b,c,d^	24.2 ^a,b,c,d^
**Jam 5**		40.9 ^a,b,c,d^	50.9 ^a,b,c,d^	45.5 ^a,b,c,d^	50.9 ^a,b,c,d^	16.3 ^a,d^	10.3 ^a,d^	8.1 ^a,d^	10.9 ^a,d^
**Jam 6**		52.9 ^d^	46.7 ^d^	47.4 ^d^	42.9 ^d^	13.7 ^a,b,d^	8.9 ^a,b,d^	9.7 ^a,b,d^	8.7 ^a,b,d^
**Jam 7**		49.2 ^d^	48.8 ^d^	50.6 ^d^	46.4 ^d^	18.9 ^a,b,d^	6.4 ^a,b,d^	6.6 ^a,b,d^	8.3 ^a,b,d^

Superscripts (a,b,c,d) indicate statistically significant differences (*p* < 0.05) according to Student’s *t* tests.

## Data Availability

The data presented in this study are available in this manuscript.
